# Eye-tracking-based analysis of pharmacists’ thought processes in the dispensing work: research related to the efficiency in dispensing based on right-brain thinking

**DOI:** 10.1186/s40780-024-00341-1

**Published:** 2024-05-10

**Authors:** Toshikazu Tsuji, Kenichiro Nagata, Masayuki Tanaka, Shigeru Hasebe, Takashi Yukita, Mayako Uchida, Kimitaka Suetsugu, Takeshi Hirota, Ichiro Ieiri

**Affiliations:** 1 Department of Clinical Pharmacy, Setsunan University, Osaka, Japan; 2 Department of Pharmacy, Kyushu University Hospital, Fukuoka, Japan; 3 Department of Education and Research Center for Pharmacy Practice, Faculty of Pharmaceutical Sciences, Doshisha Women’s College of Liberal Arts, Kyoto, Japan

**Keywords:** Eye-tracking method, Thought process, Dispensing complexity, Comparative models, Right-brain thinking

## Abstract

**Background:**

Pharmacists should be aware of their thought processes in dispensing work, including differences in the dispensing complexities owing to different drug positions in the left, center, and right areas. Dispensing errors associated with “same-name drugs (a pair of drugs with the same name but a different ingredient quantity)” are prevalent and often negatively affect patients. In this study, using five pairs of comparative models, the gaze movements of pharmacists in dispensing work were analyzed using an eye-tracking method to elucidate their thought processes.

**Methods:**

We prepared verification slides and displayed them on a prescription monitor and three drug rack monitors. The dispensing information (drug name, drug usage, location display, and total amount) was displayed on a prescription monitor. A total of 180 drugs including five target drugs were displayed on the three drug rack monitors. Total gaze points in the prescription area, those in the drug rack area, total vertical movements between the two areas, and time required to dispense drugs were measured as the four classifications Gaze 1, Gaze 2, Passage, and Time, respectively. First, we defined the two types of location displays as “numeral combination” and “color/symbol combination.” Next, we defined two pairs of models A_1_-A_2_ (numerals) and B_1_-B_2_ (color/symbol) to compare differences between the left and right areas. Moreover, three pairs of models C_1_-C_2_ (left), D_1_-D_2_ (center), and E_1_-E_2_ (right) were established to compare differences between “numeral combination” and “color/symbol combination.”

**Results:**

Significant differences in the complexities of dispensing work were observed in Gaze 2, Passage, and Time between the models A_1_-A_2_ (A_1_<A_2_), in Gaze 2 between the models B_1_-B_2_ (B_1_>B_2_), and in Gaze 2 and Time between the models C_1_-C_2_, D_1_-D_2_, and E_1_-E_2_ (C_1_>C_2_, D_1_>D_2_, and E_1_>E_2_, respectively).

**Conclusions:**

Using the current dispensing rules, pharmacists are not good at dispensing drugs located in the right area. An effective measure for reducing the dispensing complexity is to introduce visual information in the prescription content; the utilization of the right brain facilitates reducing the complexity in the right dispensing area.

## Background

The accurate dispensing of drugs is arguably one of the most important steps in providing safe and secure medical care. Pharmacists must dispense a large number of drugs correctly and quickly within a predetermined time for patients’ medical therapy. Although the dispensing operations have become mechanized utilizing one-dose package machines in many medical institutions, pharmacists frequently encounter situations that require manual dispensing of drugs. Therefore, pharmacists need to devise more efficient dispensing methods and strive to maintain an environment that promotes safe dispensing, concurrently with the introduction of mechanical support. Because the complexity of dispensing work increases the probability of errors occurring, continuous efforts are being made in various medical institutions to prevent erroneous dispensing [1–12]. Due to continuous efforts in Kyushu University Hospital to prevent near misses and dispensing errors, the incidence rate of their patients using incorrect drugs has not exceeded 0.038% since 2006 [13–19]. However, as human beings can make mistakes, the prevention of all errors caused by pharmacists is extremely difficult. Therefore, pharmacists should aim to reduce the dispensing complexities which increase the probability of errors occurring. Consequently, pharmacists must be aware of their underlying thought processes in dispensing work, for example, differences in complexities of dispensing owing to drug positions in racks (left, center, and right areas) and the proper use of left- and right-brain functions for pinpointing drug locations.

Eye-tracking systems use sensor technology to detect and follow a person’s eye movements in real-time. The verification method in this study was based on that in previous reports [20, 21], in which we clarified the basic confirmation process of target drugs in 12 pharmacists by using an eye-tracker. These previous studies demonstrated that the dispensing process became more complicated in case of dispensing “same-name drugs” or being located in the “right side area” in the drug rack. Furthermore, we elucidated the thought processes of pharmacists in simple and complex environments, as well as the mechanisms of error occurrence using error-induction models. However, dispensing errors associated with “same-name drugs” are prevalent and often negatively affect patients.

In the present study, we investigated the differences in gaze movements between left and right areas by setting “numeral combination” or “color/symbol combination” as the display method of drug location (models A_1_-A_2_ and B_1_-B_2_, respectively), furthermore, the differences in gaze movements between “numeral combination” and “color/symbol combination” in the left, center, and right areas (models C_1_-C_2_, D_1_-D_2_, and E_1_-E_2_, respectively). Here, although the details regarding the left- and right-brain functions of human are unknown, there is a report that the right-brain thinking is closely related to both color recognition and color processing [22]. Thus, the present study introduced visual information such as colors or symbols into the location display in the prescription content in an extension of previous studies. We aimed to prove the effectiveness of using the right brain in dispensing work by analyzing the thought processes of pharmacists in various dispensing environments.

## Methods

### Verification using the eye-tracking system

Eye-tracking, a method of verifying gaze movements by detecting the corneal reflex of infrared rays, is used in various fields such as medicine, psychology, and cognitive science [23–26]. In this study, we investigated the gaze movements of pharmacists in the dispensing process using a glasses-like eye tracker (Tobii Pro Glasses 3, Tobii Technology K.K.). Gaze movements obtained by eye-tracking were mainly classified into the two categories fixation (stagnation within a 20-pixel window for a minimum of 100 ms) and saccade (quick movements of the eyeballs). Fixation and saccade were judged from recorded motion videos using dedicated analysis software (Tobii Pro Lab Analyzer, Tobii Technology K.K.).

### Target persons and drugs

The inclusion criteria for pharmacists in this study were as follows. First, an essential criterion required for accurate eye movement measurements was that pharmacists should be able to read the dispensing information displayed on the large monitors with their naked eyes or while using soft contact lenses. Second, pharmacists should have more than 18 months of dispensing experience at the Kyushu University Hospital; this was essential to maintain the quality of verification above a certain level. Finally, pharmacists should agree to participate in this study.

The target drugs used in this study were 15 pairs of same-name drugs dispensed in the hospital. Here, “same-name drug” refers to a pair of drugs with the same name (character part) but a different ingredient quantity (number part). The target drugs were tablets of famotidine OD 10 mg/20 mg, nauzelin^®^ OD 5 mg/10 mg, forxiga^®^ 5 mg/10 mg, eliquis^®^ 2.5 mg/5 mg, zolpidem 5 mg/10 mg, furosemide 20 mg/40 mg, cilostazol OD 50 mg/100 mg, atorvastatin 5 mg/10 mg, depakene^®^ R 100 mg/200 mg, and decadron^®^ 0.5 mg/4 mg, furthermore, the former drugs in five pairs of ones are as follows: losartan K 25 mg/50 mg, Topina^®^ 50 mg/100 mg, tegretol^®^ 100 mg/200 mg, belsomra^®^ 20 mg/25 mg, and thyradin^®^ S 25 µg/50 µg.

### Preparation of the verification slides

The slides used for dispensing verifications in this study were created using Microsoft PowerPoint^®^ 2016 and each dispensing verification was performed as a set of one prescription slide and three drug rack slides. Five target drugs were dispensed in each verification. The dosage and administration of each target drug were appropriate and there were no drug interactions among the five target drugs.

Regarding the content of a prescription slide, basic information consisting of patient name, age (sex), body weight, height, and creatinine clearance value was displayed at the upper side of the slide. Moreover, the dispensing information consisting of four items namely (a) drug name, (b) drug usage, (c) location display, and (d) total amount was displayed in the center of the slide. Regarding the content in each drug rack slide, a grid-type rack of 5 rows × 10–14 columns was displayed, each cell containing a name label of a drug at the bottom. A total of three drug rack slides were prepared in each verification, and the five target drugs were arranged at specified positions on them. Drugs with the same initial character and ingredient quantity (number part) were not displayed in the same row as the target drug. Additionally, the arrangement of drugs on the verification slides differed significantly from the actual dispensing state at Kyushu University Hospital. There was no adherence to either alphabetical order or order based on drug efficacy. Thus, approximately 180 drugs including the five target drugs were displayed on the three drug rack slides.

In this study, the indication method of “(c) location display” in the prescription slide was classified into two types: “numeral combination” and “color/symbol combination.” For example, “3-4-2” as the numeral combination indicated that the target drug was located on monitor-3, fourth row from the top, and second column from the left. Likewise, “➂  ” as the color/symbol combination indicated that the drug was located on monitor-3, on the blue line, and second from the left. For “color/symbol combination,” five colors (red, yellow, green, blue, and black) were used as the location display, and a colored line was shown above each row in the drug rack. The five target drugs were not located in the same row and column (x–1–1, x–2–2, x–3–3, x–4–4, or x–5–5; where “x” indicates the monitor number). Details of the dispensing information are presented in Table 1. In a series of studies as dispensing verifications, we generated 14 prescription slides and 42 drug rack slides to perform a total of seven pairs of dispensing verifications. The order of verifications was random. Notably, we only analyzed the data acquired from five of the seven pairs of verifications in this study.

**Table 1:**
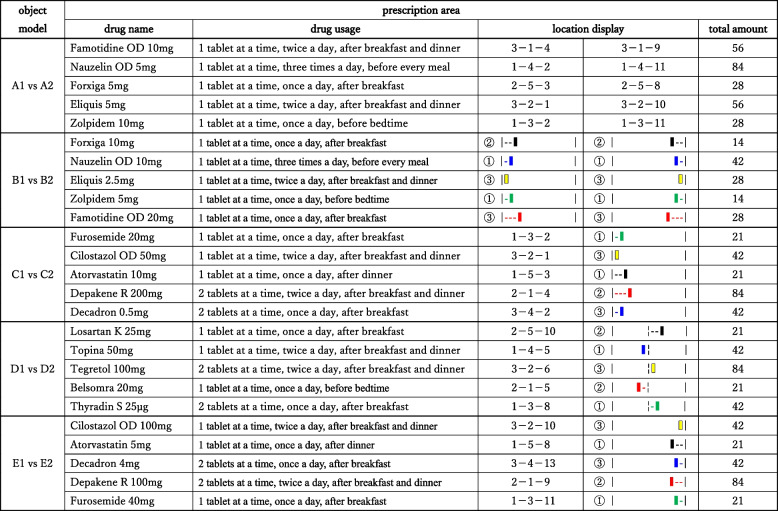
List of verification information

Object model and prescription information (drug name, drug usage, location display, and total amount) are displayed. Regarding location display, “3-4-2” as a sample “numeral combination” means that the target drug is located on monitor-3, in the fourth row from the top, and in the second column from the left. Likewise, “➂  ” as the sample “color/symbol combination” means that the drug is located on monitor-3, on the blue line, and in the second column from the left

### Verification procedure

**Fig. 1:**
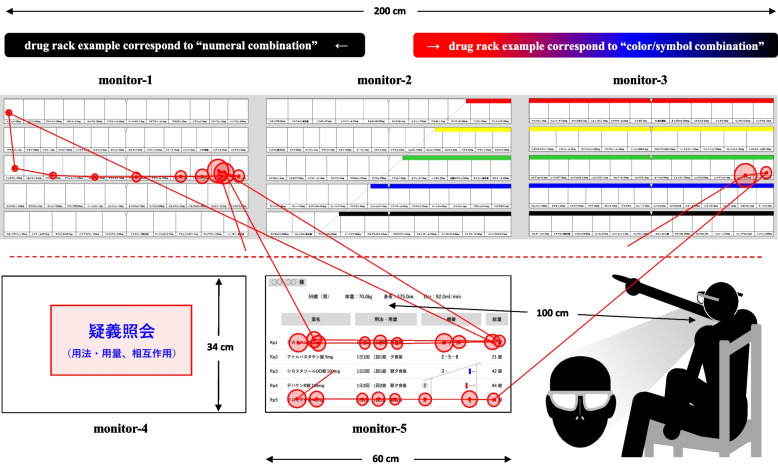
Outline of the verification process using the eye-tracking method. Gaze movements obtained by eye-tracking and analyzed using Tobii Pro Lab Analyzer were mainly classified into the two categories fixation (stagnation for a certain time) and saccade (quick movements of the eyeballs). We analyzed a series of dispensing processes by showing the prescription (length 34 cm × width 60 cm) and drug rack (length 34 cm × width 200 cm) areas. The red dotted line in the figure represents the boundary between the two areas, the left side from the center oblique line on monitor-2 corresponds to the drug rack example using the display method of “numeral combination,” while the right side from the line corresponds to the “color/symbol combination.” The center point in the circle, the size of the circle, and the line between the center points of circles indicate the gazing point, gazing time, and gaze movement, respectively. A pharmacist wearing an eye tracker was seated on a chair 100 cm from monitor-5 and performed several pairs of dispensing verifications in random order

An outline of the verification task using the eye-tracking method is shown in Fig. 1. We connected five notebook computers to 27-inch monitors (monitor-1, -2, -3, -4, and -5) to operate the slides. The drug rack area (length 34 cm × width 200 cm) on monitor-1, -2, and -3 was on the upper stage, and the prescription area (length 34 cm × width 60 cm) on monitor-5 directly below monitor-2 was on the lower stage. Monitor-4 for the prescription inquiry was arranged to the left of monitor-5. A pharmacist wearing an eye tracker was seated on a chair 100 cm from monitor-5 to read the prescription slide. By showing the drug rack area (monitor-1, -2, and -3) and the prescription area (monitor-5) simultaneously, we could investigate the gaze movements of pharmacists during the dispensing process. Using Tobii Pro Lab Analyzer with the recorded motion video, we could also assess several categories, such as gazing point (center point in the circle), gazing time (size of the circle), and gaze movement (line between center points of circles).

To ensure the accuracy of the eye tracker, we performed gaze calibration with each pharmacist before conducting the verification experiments. To get used to the verification process, pharmacists were allowed to practice with several training slides in advance. Smooth dispensing as usual was prioritized in this verification task, and if a pharmacist noticed a mistake in the dispensing process, they were allowed to correct the mistake immediately. Furthermore, if the pharmacist determined that there was an issue with the prescription content, they would point at the monitor-4 for further inquiry regarding the prescription. We analyzed a series of verification processes, from confirming the dispensing information in the prescription area to pinpointing the five target spots in the drug rack area. The main steps for dispensing verifications were as follows:

1. A pharmacist gazed at a given position.
2. An assistant switched to a prescription slide and three drug rack slides simultaneously when the “Next” signal was indicated by the pharmacist.
3. The pharmacist read out “total amount” of a target drug while pinpointing the target spot and repeated this process a total of five times.
4. The assistant switched to a rest slide when the “Next” signal was indicated by the pharmacist.
5. The verifications, using 14 prescription and 42 drug rack slides, were repeated with voluntary breaks.

### Definition of the five paired models

**Fig. 2:**
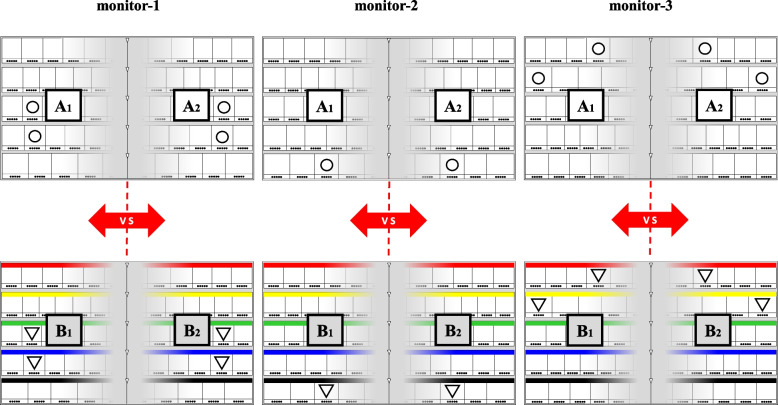
Arrangement of the five target drugs in two pairs of models A_1_-A_2_ (upper side) and B_1_-B_2_ (lower side). The location displays of the five target drugs in the two pairs of models A_1_-A_2_ and B_1_-B_2_ are indicated using the “numeral combination” and “color/symbol combination” methods, respectively. These five target drugs in each pair of models (A_1_-A_2_ and B_1_-B_2_) are the same, but their locations are arranged left-right symmetrically regarding the center line (left vs right area). The location spots of the five target drugs in models A_1_-A_2_ and B_1_-B_2_ are shown as white circles (○) and white triangles (▽), respectively

First, we set up two pairs of models (A_1_-A_2_ and B_1_-B_2_; Fig. 2) to compare the difference in gaze movements between the left and right areas. The location display of the five target drugs in models A_1_-A_2_ was expressed by a numeral combination, whereas that in models B_1_-B_2_ was expressed by a color/symbol combination. The five target drugs in each pair of models were the same, but their locations differed in that they were arranged left-right symmetrically regarding the center line.

**Fig. 3:**
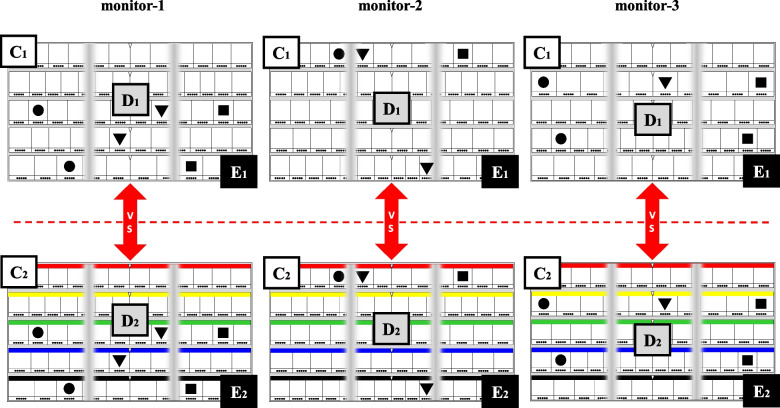
Arrangement of the five target drugs in three pairs of models C_1_-C_2_ (left area), D_1_-D_2_ (center area), and E_1_-E_2_ (right area). The location displays of the five target drugs in the three pairs of models C_1_-C_2_, D_1_-D_2_, and E_1_-E_2_ are indicated using the “numeral combination” (upper side) and “color/symbol combination” (lower side) method. These five target drugs and their locations in each pair of models (C_1_-C_2_, D_1_-D_2_, and E_1_-E_2_) are the same. The location spots of the five target drugs in models C_1_-C_2_, D_1_-D_2_, and E_1_-E_2_ are shown as black circles (●), triangles (▼), and squares (■), respectively

Second, we set up three pairs of models (C_1_-C_2_, D_1_-D_2_, and E_1_-E_2_; Fig. 3) to compare the difference in gaze movements between the location displays in “numeral combination” and “color/symbol combination” in the left, center, and right areas. The five target drugs and their locations were the same in each pair of models, but their location display methods differed in being “numeral” or “color/symbol” combinations. A summary of the two pairs (A_1_-A_2_ and B_1_-B_2_; Fig. 2) and three pairs (C_1_-C_2_, D_1_-D_2_, and E_1_-E_2_; Fig. 3) of models are given below.

- Model A_1_-A_2_: comparison between the left and right areas by the location display using “numeral combination.”
- Model B_1_-B_2_: comparison between the left and right areas by the location display using “color/symbol combination.”
- Model C_1_-C_2_: comparison between “numeral combination” and “color/symbol combination” in the left area.
- Model D_1_-D_2_: comparison between “numeral combination” and “color/symbol combination” in the center area.
- Model E_1_-E_2_: comparison between “numeral combination” and “color/symbol combination” in the right area.

### Verification items and classifications

To dispense a target drug in the verification task accurately, a pharmacist needs to visually recognize the four items (a) drug name, (b) drug usage, (c) location display, and (d) total amount in the prescription area and specify exactly a target spot in the drug rack area. Furthermore, the pharmacist also needs to move the visual line up and down as (e) vertical movement between two areas. Here, a total of gaze points in the prescription area, those in the drug rack area, a total of vertical movements passing through a boundary between two areas, and the time required to dispense the five target drugs were measured in the four classifications Gaze 1, Gaze 2, Passage, and Time. We calculated the differences in gaze movements between the left and right areas according to each location display of “numeral combination” or “color/symbol combination” (models A_1_-A_2_ and B_1_-B_2_, respectively), as well as those between the location displays of “numeral combination” and “color/symbol combination” in the left, center, and right areas (models C_1_-C_2_, D_1_-D_2_, and E_1_-E_2_, respectively).

- Gaze 1: A total of gaze points in four items of (a), (b), (c), and (d) in the prescription area.
- Gaze 2: A total of gaze points including five target spots in the drug rack area.
- Passage: A total of (e) vertical movements between the prescription and the drug rack areas.
- Time: A length of time required to dispense five target drugs.

### Calculation of reconfirmation frequency per target drug in three areas (left, center, and right)

To analyze the differences in dispensing complexities between the display methods “numeral combination” and “color/symbol combination” in three areas (left, center, and right), it is necessary to compare the reconfirmation frequency of items (a)–(e) per target drug between each pair of models C1-C2, D1-D2, and E1-E2.

Thus, we first defined the “essential number” as the minimum checks of items (a)–(e) required for dispensing a target drug as follows: (a’) 2 points, (b’) 3 points, (c’) 1 or 2 points, (d’) 1 point, and (e’) 2 times. Concerning the display method “color/symbol combination,” the number of gaze positions varied depending on the drug location. For instance, the essential number of the (c) location display was one in the left area but two in both the center and right areas. Using these definitions, we then calculated the average reconfirmation frequencies (a”)–(e”) by subtracting the essential numbers (a’)–(e’) from the average gaze values (a)–(e) per target drug. Following this new definition, the average reconfirmation frequencies (a”)–(e”) per target drug were calculated for the three pairs of models.

### Data analysis

Using the gaze category (fixation, saccade) data, we analyzed the gaze frequency in the prescription area, that in the drug rack area, the number of vertical movements between the prescription and drug rack areas, and the length of the dispensing time. Data were presented as the mean ± standard deviation of participants, and differences were analyzed using the paired *t*-test. *P* value of <0.05 was considered statistically significant; *P* values of <0.01 and <0.001 were considered highly significant. The paired *t*-test was performed using JMP Pro 15 statistical software.

## Results

### Basic information about the participating pharmacists and the verification data

Twenty-two pharmacists (9 men and 13 women) with an average age of 30.1±5.9 years participated in this study. Among them, 11 pharmacists had less than 3 years of dispensing experience (4 men and 7 women; age, 25.3±0.5 years); the other 11 pharmacists had more than 5 years of dispensing experience (5 men and 6 women; age, 34.9±4.6 years).

### Comparison of gaze movements between the left and right areas according to the display type of drug location

**Fig. 4:**
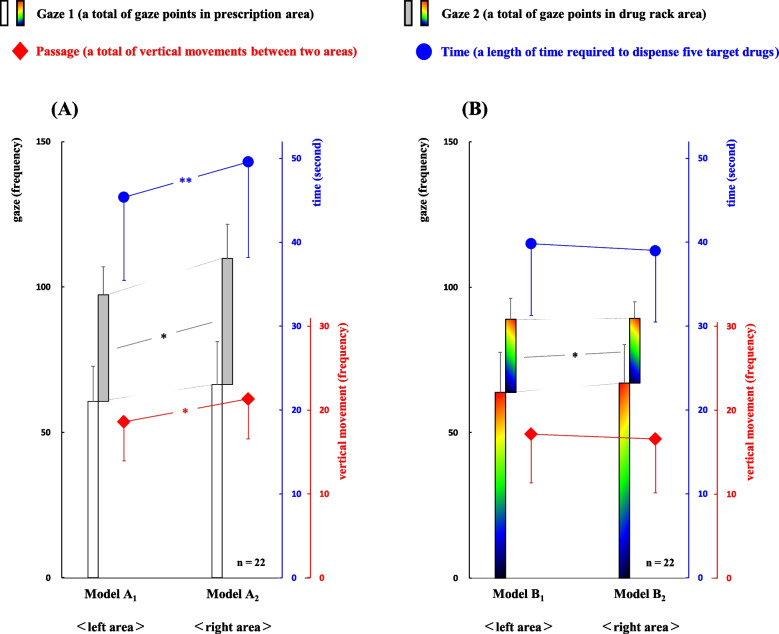
Comparison of gaze movements between left and right areas according to the display type of drug location. The relationships of gaze movements between each pair of models A_1_-A_2_ and B_1_-B_2_ are shown. Significant differences between the models A_1_-A_2_ can be observed in Gaze 2, Passage, and Time (*P*=0.032, *P*=0.016, and *P*<0.01, respectively), and those between models B_1_-B_2_ can be observed in Gaze 2 (*P*=0.037). *P<0.05, **P<0.01 using the paired *t*-test

To clarify the difference in gaze movements between the left and right areas, we analyzed the eye-tracker data from two pairs of models (A_1_-A_2_ and B_1_-B_2_; Fig. 4). Models A_1_-A_2_ were left-right symmetrical versions using the display method “numeral combination,” whereas models B_1_-B_2_ were those using the display method “color/symbol combination.”

Significant differences between the models A_1_-A_2_ were observed in the three classifications Gaze 2, Passage, and Time (*P*=0.032, *P*=0.016, and *P*<0.01, respectively). By contrast, only the classification Gaze 2 was significantly different between the models B_1_-B_2_ (*P*=0.037).

- Model A_1_: Gaze 1, 60.7±12.0; Gaze 2, 36.7±9.5; Passage, 18.6±4.7; Time, 45.4±9.9
- Model A_2_: Gaze 1, 66.4±14.8; Gaze 2, 43.5±11.7; Passage, 21.3±4.8; Time, 49.6±11.4
- Model B_1_: Gaze 1, 63.8±13.8; Gaze 2, 25.1±7.1; Passage, 17.1±5.8; Time, 39.9±8.5
- Model B_2_: Gaze 1, 67.0±13.3; Gaze 2, 22.3±5.8; Passage, 16.6±6.4; Time, 39.0±8.5

### Comparison of gaze movements between two display types of drug location in three areas (left, center, and right)

**Fig. 5:**
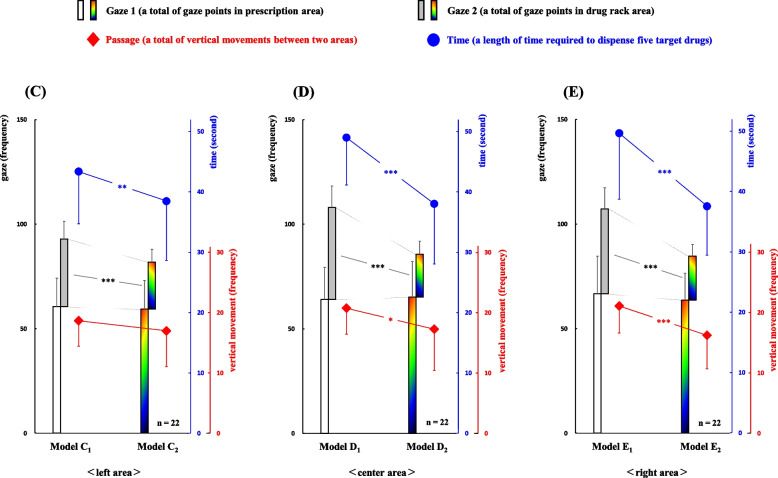
Comparison of gaze movements between two display types of drug location in three areas (left, center, and right). The relationships of gaze movements between each pair of models C_1_-C_2_, D_1_-D_2_, and E_1_-E_2_ are shown. Significant differences between the models C_1_-C_2_ can be observed in Gaze 2 and Time (*P*<0.001 and *P*<0.01, respectively). The models D_1_-D_2_ significantly differ in Gaze 2, Passage, and Time (*P*<0.001, *P*=0.023, and *P*<0.001, respectively). Furthermore, differences between the models E_1_-E_2_ can be observed in Gaze 2, Passage, and Time (*P*<0.001, *P*<0.001, and *P*<0.001, respectively). *P<0.05, **P<0.01, ***P<0.001 using the paired *t*-test

To clarify the difference in gaze movements between the display methods “numeral combination” and “color/symbol combination” in the left, center, and right areas, we analyzed movement data using three pairs of models (C_1_-C_2_, D_1_-D_2_, and E_1_-E_2_; Fig. 5).

Significant differences between the models C_1_-C_2_ were observed in the two classifications Gaze 2 and Time (*P*<0.001 and *P*<0.01, respectively). Moreover, significant differences between the models D_1_-D_2_ were observed in the three classifications Gaze 2, Passage, and Time (*P*<0.001, *P*=0.023, and *P*<0.001, respectively). Likewise, significant differences between the models E_1_-E_2_ were observed in the three classifications Gaze 2, Passage, and Time (*P*<0.001, *P*<0.001, and *P*<0.001, respectively).

- Model C_1_: Gaze 1, 60.6±13.5; Gaze 2, 32.4±8.4; Passage, 18.6±4.2; Time, 43.4±8.6
- Model C_2_: Gaze 1, 59.4±13.7; Gaze 2, 22.5±6.0; Passage, 17.0±5.9; Time, 38.5±9.8
- Model D_1_: Gaze 1, 64.0±15.4; Gaze 2, 44.0±10.3; Passage, 20.7±4.3; Time, 49.0±7.9
- Model D_2_: Gaze 1, 65.1±17.1; Gaze 2, 20.5±6.2; Passage, 17.3±6.8; Time, 38.1±10.0
- Model E_1_: Gaze 1, 66.8±17.9; Gaze 2, 40.5±10.0; Passage, 21.1±4.5; Time, 49.7±10.9
- Model E_2_: Gaze 1, 63.6±12.9; Gaze 2, 21.0±5.7; Passage, 16.2±5.6; Time, 37.6±8.1

### Comparison of reconfirmation frequency per target drug in three areas (left, center, and right)

**Fig. 6:**
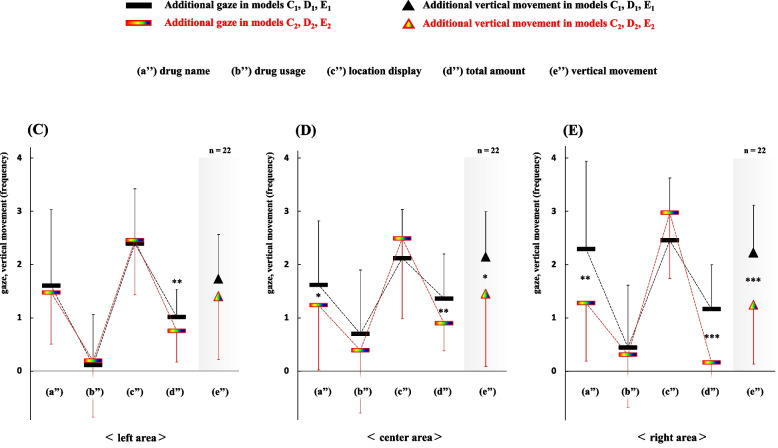
Comparison of reconfirmation frequency per target drug between two display types of drug location in three areas (left, center, and right). The relationships of reconfirmation frequency per target drug between each pair of models C_1_-C_2_, D_1_-D_2_, and E_1_-E_2_ are shown. A significant difference between the models C_1_-C_2_ (left area) can be observed in the item (d”) (*P*<0.01). The models D_1_-D_2_ (center area) significantly differ in the items (a”), (d”), and (e”) (*P*=0.043, *P*<0.01, and *P*=0.023, respectively). Furthermore, differences between the models E_1_-E_2_ (right area) can be observed in the items (a”), (d”), and (e”) (*P*<0.01, *P*<0.001, and *P*<0.001, respectively). *P<0.05, **P<0.01, ***P<0.001 using the paired *t*-test

To elucidate the differences in dispensing complexities between the display methods “numeral combination” and “color/symbol combination” in three areas (left, center, and right), we conducted an analysis of the reconfirmation frequency of items (a)–(e) per target drug between each pair of models (C_1_-C_2_, D_1_-D_2_, and E_1_-E_2_; Fig. 6).

A significant difference in the complexities of dispensing tasks was observed in item (d”) between models C_1_-C_2_ located in the left area (C_1_>C_2_, *P*<0.01). Moreover, significant differences were observed in items (a”), (d”), and (e”) between models D_1_-D_2_ located in the center area (D_1_>D_2_, *P*=0.043, *P*<0.01, and *P*=0.023, respectively), and between models E_1_-E_2_ located in the right area (E_1_>E_2_, *P*<0.01, *P*<0.001, and *P*<0.001, respectively).

- Model C_1_: (a”), 1.6±1.4; (b”), 0.1±1.0; (c”), 2.4±1.0; (d”), 1.0±0.5; (e”), 1.7±0.8
- Model C_2_: (a”), 1.5±1.0; (b”), 0.2±1.1; (c”), 2.5±1.0; (d”), 0.8±0.6; (e”), 1.4±1.2
- Model D_1_: (a”), 1.6±1.2; (b”), 0.7±1.2; (c”), 2.1±0.9; (d”), 1.4±0.8; (e”), 2.1±0.9
- Model D_2_: (a”), 1.2±1.2; (b”), 0.4±1.2; (c”), 2.5±1.5; (d”), 0.9±0.5; (e”), 1.5±1.4
- Model E_1_: (a”), 2.3±1.7; (b”), 0.4±1.2; (c”), 2.5±1.2; (d”), 1.2±0.8; (e”), 2.2±0.9
- Model E_2_: (a”), 1.3±1.1; (b”), 0.3±1.0; (c”), 3.0±1.2; (d”), 0.2±0.5; (e”), 1.2±1.1

## Discussion

In this study, we aimed to elucidate the thought processes of pharmacists under various dispensing environments using an eye-tracking system. Accordingly, we analyzed the differences in gaze movements between the left and right areas by setting up two pairs of models (A_1_-A_2_ and B_1_-B_2_) using as the location displays “numeral combination” and “color/symbol combination,” respectively. Moreover, the differences in gaze movements between “numeral combination” and “color/symbol combination” in the left, center, and right areas were assessed in three additional pairs of models (C_1_-C_2_, D_1_-D_2_, and E_1_-E_2_, respectively). The results not only demonstrated that pharmacists are not good at dispensing drugs located in the right area when using the current dispensing method (numeral combination) but also that the introduction of visual information such as colors or symbols into the prescription content reduces the dispensing complexity in the right area. Here, the display method of “color/symbol combination” enables the representation of vertical direction as “color” and horizontal direction as “symbol” in the drug rack. Particularly, both recognition and processing of “color” have been reported to be closely related to right-brain thinking [22]. In summary, these results suggest that pharmacists can engage in dispensing work more effectively and safely by utilizing right-brain thinking.

First, significant differences in the complexities of dispensing work between the models A_1_-A_2_ were observed in the classifications Gaze 2, Passage, and Time (A_1_<A_2_, *P*=0.032, *P*=0.016, and *P*<0.01, respectively), which indicates that the dispensing work using the display method “numeral combination” was more complex in the right area (Fig. 4-A). The location display using numeral combinations (e.g., 3-4-2, 2-5-3, 1-3-4) plays a role in the “translation code” which converts the numerical information into position information. The calculation ability, such as memory, conversion, and storage of the numerical information, is essential to accurately pinpoint the position of the target drug, but it is quite different from other thought processes such as pharmaceutical judgment regarding the prescription content. Moreover, the pharmacist has only one way to confirm the drug location in L-shaped order (from upper to lower, from left to right), when following the display method “numeral combination.” In essence, the 3rd number in the “numeral combination”, which represents the horizontal drug position, increases as the drug position shifts from left to right. This is because the starting point of the viewpoint movement is only at the left end of the drug rack, which complicates the dispensing process in the right area in the case of “numeral combination.” Here, the memory capacity of a human is limited; for example, the amount of information that can be kept in short-term memory is 7±2 items, and subsequent research suggested the amount of information to be limited to 4±1 items. Moreover, memory content is erased over time or by interfering information [27–29]. Thus, it is unsurprising that pharmacists need to think more deeply when the numeric combination becomes more complex. What is important here is that the increased complexity of pinpointing the target drugs in the right area led to a significant increase in vertical movements of visual line and ultimately a significant increase in dispensing time.

Second, a significant difference in the complexity of dispensing work between the models B_1_-B_2_ was only observed in the classification Gaze 2 (B_1_>B_2_,* P*=0.037). This indicates that when using the display method “color/symbol combination,” the dispensing complexity was almost equivalent for the left and right areas (Fig. 4-B). In summary, the display method of “color/symbol combination” allows for the vertical drug position to be represented as “color” and the horizontal drug position as “symbol” on the drug rack. Moreover, it enables the horizontal drug position to be accessed from both the left and right directions with the same level of efficiency by recognizing the horizontal position through the symbol code. Consequently, this result suggests that it was easy for pharmacists to pinpoint the target spots even in the right area by introducing visual information such as color/symbol into the location display in the prescription information. Thus, the introduction of visual information into prescription content reduces the complexity of dispensing work in the right area, suggesting that the utilization of right-brain thinking may eliminate left-right differences in dispensing complexity caused by the locations of drugs.

Third, significant differences in the complexities of dispensing work between each pair of models C_1_-C_2_, D_1_-D_2_, and E_1_-E_2_ were observed in the classifications Gaze 2 and Time in the left area (C_1_>C_2_, *P*<0.001 and *P*<0.01, respectively), Gaze 2, Passage, and Time in the center area (D_1_>D_2_, *P*<0.001, *P*=0.023, and *P*<0.001, respectively) and Gaze 2, Passage, and Time in the right area (E_1_>E_2_, *P*<0.001, *P*<0.001, and *P*<0.001, respectively). These results suggest that the dispensing work using the “numeral combination” method was more complex than that using the “color/symbol combination” method in each of the areas left, center, and right. Particularly, the significant differences regarding Passage (a total of vertical movements between the prescription and the drug rack areas) between each pair of models C_1_-C_2_, D_1_-D_2_, and E_1_-E_2_ became relatively more remarkable as shifting from the left area toward the right area. By contrast, the classification Gaze 1 (a total of gaze points in the prescription area) was not significantly different between each pair of models (left, center, and right; Fig. 5). Thus, the detailed relationship between Gaze1 and Passage could not be clarified only from these results.

Finally, concerning the reconfirmation frequency of items (a)–(e) per target drug between each pair of models, a significant difference in the complexities of dispensing work was observed in item (d”) between models C_1_-C_2_ located in the left area (C_1_>C_2_, *P*<0.01). Additionally, significant differences were observed between models D_1_-D_2_ in items (a”), (d”), and (e”) located in the center area (D_1_>D_2_, *P*=0.043, *P*<0.01, and *P*=0.023, respectively) and between models E_1_-E_2_ located in the right area (E_1_>E_2_, *P*<0.01, *P*<0.001, and *P*<0.001, respectively). These data suggest that the difference in dispensing complexity between each pair of models C_1_-C_2_, D_1_-D_2_, and E_1_-E_2_ (left, center, and right) increased as the drug positions shifted from the left toward the right area. Furthermore, the change in significant differences between (a”) drug names were closely linked to those between (e”) vertical movements in all three areas. Notably, the reconfirmation frequency of an item (c) location display using the “color/symbol combination” was higher than that using the “numeral combination” in both the center and right areas (D1<D2, E1<E2). As the reason for this, it is considered that pharmacists had to divide the item (c) location display into two parts and check them separately in the “color/symbol combination,” as the distance between two symbols increased in both the center and right areas. Undoubtedly, the significant increase in vertical movements of visual line between the prescription and the drug rack areas reflected distinctly the complexity of the dispensing process, this analysis revealed that the reconfirmation of (a) drug name which was so frequent as to cross the boundary had a significant impact on the increase in (e) vertical movement. Furthermore, these results suggest that the memory capacity of a pharmacist is exceeded one’s limit easier as the dispensing work is more complicated because the storage capacity of human short-term memory is not very large [26–28]. In summary, when following the display method “numeral combination” in the dispensing work, pharmacists needed more frequent checks for uncertain or forgotten items as their memory became more unreliable owing to the increased complexity of the dispensing work. By contrast, when following the display method “color/symbol combination,” it was not necessary for the pharmacist to convert the numerical information into the position information, furthermore, they could approach the drug location from several directions (upper, lower, left, right, and center).

Over the last few decades, the mechanization of dispensing operations, such as one-dose package machines, has spread through medical institutions to improve dispensing efficiency. In recent years, the use of drug barcodes has been widely introduced to prevent dispensing errors, but this creates several other problems, such as a longer time required for dispensing work, not reading the barcodes cut from the press-through package (PTP) sheets, and not preventing counting errors. Therefore, the experience and skills of pharmacists are still required to dispense drugs efficiently and quickly in real-world situations. Considering the results of the present study, it may also be important for pharmacists to take measures for the practical use of right-brain thinking such as introducing visual information into prescription content. Here, if it is true that pharmacists are not very good at dispensing drugs located on the right side of drug racks, no effective measure can improve the complications of dispensing work in such a situation because half of all drugs are stored in the right area. As such, pharmacists should make efforts to devise more efficient methods in dispensing work such as introducing visual information, in addition to maintaining the safe dispensing environments.

This study has some limitations. First, the utilization of colors in the display method “color/symbol combination” is unfit for pharmacists who are color-blind; thus, the parallel display of a “numeral combination” might be required for practical purposes. Second, available colors or symbols might be restricted as dispensing information, and the display method used in this study cannot always be applied to electronic medical charts in all medical institutions. Third, although the display method “color/symbol combination” can be applied to dispensing work with grid-type racks, it is difficult to put it into practical use in drawer-type racks. Furthermore, it is necessary to verify whether or not this result leads to the improvement of efficiency in actual dispensing work. However, to the best of our knowledge, this is the first study to adopt visual information such as colors or symbols to the current dispensing rules and to evaluate the thought processes of more than 20 pharmacists using an eye-tracking system. Therefore, the findings of this study might serve as a reference for pharmacists in other facilities as it proved the usefulness of introducing visual information based on right-brain thinking of pharmacists.

## Conclusions

When pharmacists follow the “numeral combination” display method for drug locations under the current dispensing rules, they seem to be not good at dispensing drugs located in the right area of drug racks. Therefore, we analyzed the thought processes of 22 pharmacists in various dispensing environments to prevent complications of dispensing work in the right area. The introduction of visual information into prescription content was an effective measure for reducing dispensing complexity. In other words, the utilization of the right brain in dispensing processes enabled the reduction of dispensing complexity in the right area. Thus, pharmacists should devise more efficient methods in dispensing work along with mechanical support in the future, which will further improve medical safety.

## Data Availability

All data generated or analyzed during this study are included in this published article.
